# The Acid-Buffered Engineered Gel Promotes In Vitro Cutaneous Healing and Fights Resistant Bacteria in Wounds

**DOI:** 10.3390/pharmaceutics16111484

**Published:** 2024-11-20

**Authors:** Fatima Abid, Emmeline Virgo, Tahlia Louise Kennewell, Riya Khetan, Hanif Haidari, Zlatko Kopecki, Yunmei Song, Sanjay Garg

**Affiliations:** 1 Centre for Pharmaceutical Innovation, Clinical and Health Sciences, University of South Australia, Adelaide, SA 5000, Australia; fatima.abid@mymail.unisa.edu.au (F.A.); riya.khetan@mymail.unisa.edu.au (R.K.); may.song@unisa.edu.au (Y.S.); 2 Future Industries Institute, University of South Australia, Mawson Lakes, SA 5095, Australia; virgoemmeline@gmail.com (E.V.); tahlia.kennewell@unisa.edu.au (T.L.K.); hanif.haidari@unisa.edu.au (H.H.); zlatko.kopecki@unisa.edu.au (Z.K.)

**Keywords:** wound healing, wound dressing, acid-buffered gel, wound infection

## Abstract

**Background**: Treatment of cutaneous wound infections is becoming a major clinical challenge due to the growing problem of antimicrobial resistance associated with existing wound treatments. Two prevalent pathogens in wound infections, *Staphylococcus aureus* (*S. aureus*) and *Pseudomonas aeruginosa* (*P. aeruginosa*), continue to present a serious challenge, underscoring the critical need for new therapeutic alternatives. **Methods**: Novel alginate acid-buffered gels (ABF-1, ABF-2, and ABF-3) were developed using a combination of organic acids in various concentrations and buffered at a pH of 4.5. The acid-buffering capacity of the gels was evaluated against sodium hydroxide solution and simulated wound fluid (SWF) at different wound pHs, mimicking infected and non-infected wound environments. The in vitro antibacterial activity was assessed against resistant bacterial strains (Gram-positive and Gram-negative) using a microdilution method and wound biofilm assay. The rheological properties and cell viability of the gels were evaluated and the gel showing positive cell viability was further investigated for healing ability using an in vitro wound scratch assay. **Results**: The gels showed promising in vitro antibacterial activity against *Staphylococcus epidermidis*, *S. aureus*, and *P. aeruginosa*. Gels with higher acid concentrations (ABF-1 and ABF-2) were highly effective in reducing the bacterial load in chronic biofilms of *S. aureus* and *P. aeruginosa*, while the gel with a lower acid concentration (ABF-3) showed positive effects on the viability of skin cells (over 80% cells viable) and for promoting wound closure. All three gels demonstrated excellent acid-buffering capabilities. **Conclusions**: The acid-buffered gels demonstrate promising in vitro antibacterial effects, indicating their potential for enhancing wound healing.

## 1. Introduction

Wound infections are considered a global health problem, affecting millions of individuals and placing an overwhelming strain on healthcare systems worldwide. They are associated with life-threatening complications attributed to increased microbial resistance leading to prolonged hospitalizations, increased healthcare costs, and severe impacts on quality of life [1,2,3,4]. In response to the global multidrug resistance crisis, the World Health Organization published a report focusing on the need for new antibiotics to treat resistant bacterial infections [5]. In the United States, approximately 23,000 deaths were reported from resistant bacterial infections in 2013, with 11,000 annual deaths resulting from wound and skin infections [6]. It is known that all wound types are colonized by microbes, including the commensal bacteria that naturally reside on the skin surface. However, the risk of infection is largely determined by the extent of skin integrity loss, as well as the bacterial load and virulence of the invading microorganisms [7]. The bacterial species predominantly found in clinical wounds *include Staphylococcus aureus* (*S. aureus*), *Pseudomonas aeruginosa* (*P. aeruginosa*), *Escherichia coli* (*E. coli*), *Corynebacterium* species, and *Proteus* species [8]. As microbial colonization in the wound bed increases, the risk of developing an infection also rises [9]. Among the current investigations, the presence of bacterial biofilms in chronic wounds has been associated with clinically difficult-to-treat wound infections [10]. Notably, biofilms formed by *P. aeruginosa* and *S. aureus* are associated with greater resistance to antimicrobial treatments, often requiring higher doses for successful therapeutic outcomes [10,11,12,13,14]. This presents a significant challenge in healthcare settings, resulting in high morbidity and mortality rates [15,16,17]. As a result, there is a growing demand for alternative, effective therapeutic approaches to combat drug-resistant microbes and successfully manage biofilms in wound infections.

The pH of the wound plays the predominant role in infection progression, with alkaline pHs known for promoting their growth while acidic pHs are detrimental to their survival [18,19,20,21,22,23]. Studies investigating wound microenvironments have shown that acidic environments accelerate wound healing by enhancing cellular migration, optimizing enzyme activity [24,25,26,27,28], and suppressing the growth of *S. aureus* and *E. coli* [29]. Additionally, maintaining a low pH environment has been demonstrated to disrupt biofilm formation, weaken existing biofilms, and enhance the efficacy of antibacterial treatments [30,31,32,33].

Several studies have explored the use of organic acids due to their biocompatibility and effectiveness in modulating pH in wound care to promote healing and inhibit bacterial proliferation, including a formulation developed by Sim et al. (2023) demonstrating an enhanced wound regeneration with significant antibacterial effects against Gram-positive bacteria using citric acid buffered at a pH of 4 [29]. A few other studies demonstrated similar effects using acetic acid by changing ionic strengths, finding that higher ionic strength exhibits more pronounced antibacterial and biofilm-inhibitory effects [34,35]. This finding highlights the potential to tailor acid-based treatments to maximize their therapeutic efficacy, particularly in targeting antibiotic-resistant bacteria embedded in biofilms. By disrupting biofilm integrity and reducing bacterial load, these formulations can enhance wound healing and reduce the risk of chronic infection. Regrettably, there is a notable lack of research focused on the development of gel formulations that are buffered to an acidic pH to treat wound infections. Even though citric acid [29,36,37,38,39], acetic acid [40,41,42,43,44,45], and boric acid [46] have previously been investigated individually for healing infected wounds, no preparation has been prepared previously to investigate the effect of their combination on the healing of infected wounds. Furthermore, to the best of our knowledge, no such formulation has yet been tested for its efficacy against bacterial wound biofilms.

The current study focused on developing a novel formulation designed to treat wound infections by exploiting the antibacterial, anti-biofilm, and wound-healing properties of an acid-buffered gel. To achieve this, various alginate-buffered gels were developed using a combination of organic acids in different concentrations and characterized by their viscosity and buffering capacity against sodium hydroxide solution and simulated wound fluid (SWF) maintained at different pH ranges. The gels’ antibacterial and wound biofilm properties were then tested against *S. aureus* and *P. aeruginosa* using in vitro assays showing promising antibacterial properties. Meanwhile, the in vitro biocompatibility was also assessed using cell viability and wound scratch assays with fibroblasts (HFFs) and keratinocytes (HaCaTs).

## 2. Materials and Methods

### 2.1. Materials

Alginic acid derived from brown algae (MW = 176.10 g/mol), citric acid (MW = 210.14 g/mol; ≥99.0%), potassium hydrogen phthalate (KHP), L-glutamine, and penicillin–streptomycin were ordered from Sigma-Aldrich (Sydney, New South Wales (NSW), Australia). Boric acid (MW = 61.83 g/mol; 99.5%) was purchased from Optigen Scientific (Adelaide, South Australia (SA), Australia), while sodium hydroxide (NaOH) pallets and glacial acetic acid (analytical grade) were ordered from Chem Supply (Adelaide, SA, Australia). Ultrapure water with 18.2 MΩ (Millipore) was used in all syntheses and preparations. Fetal calf serum (FCS), maximum recovery diluent (MRD), high glucose Dulbecco’ modified eagle medium (DMEM), fetal bovine serum (FBS), Gibco™ N-2-hydroxyethyl piperazine-N-2-ethane sulfonic acid (HEPES), Gibco™ MEM NEAA (non-essential amino acids), and Dulbecco’ phosphate-buffered saline (PBS) were purchased from Thermo Fisher Scientific (Melbourne, Victoria (VIC), Australia). Dimethyl sulfoxide (DMSO) was ordered from Merck (Melbourne, VIC, Australia) and 3-(4,5-Dimethylthiazol-2-yl)-2,5-diphenyltetrazolium bromide (MTT) was ordered from Life Technologies Australia (Melbourne, VIC, Australia). Tryptone soy broth (TSB) and tryptic soy agar (TSA) were ordered from Thermo Fisher Scientific, Oxoid, and Bacto^TM^ brain heart infusion (BHI) was purchased from Bacto laboratories (Sydney, NSW, Australia). Xanthan gum NF was obtained from Letco Medical, LLC (Decatur, AL, USA).

### 2.2. Development of Acid-Buffered Gel

#### 2.2.1. Standardization of Sodium Hydroxide Solution

A total of 5 g of primary standard (KHP) was weighed into 100 mL Erlenmeyer flasks. To each flask, 75 mL of distilled water was added, followed by two drops of phenolphthalein indicator. Then, titration was performed against 1N NaOH until a pink color was observed. This titration was repeated in triplicate.

#### 2.2.2. Preparation of Acid-Buffered Solution

The acid-buffered solutions were composed of different concentrations of a combination of citric acid, acetic acid, and boric acid with alginic acid. Studies have found concentrations of 1–5%, and 0.16–5% are safe and effective antibacterial concentrations for citric acid [29,36,37,38,39] and acetic acid [40,41,42,43,44,45], respectively, significantly reducing bacterial counts in chronic wounds, whereas boric acid has been found to be effective in healing wounds at concentrations of 2–3% [46]. Therefore, in this research, formulations were developed composed of acids in various concentrations (Table 1) previously reported to be effective and safe for biological activities.

For the preparation of an acid-buffered solution, using the method adopted from one of our patented acid-buffered formulations [47], known amounts of acids were first dissolved in 5 mL of MilliQ water under constant mixing at 25 °C to form a clear solution. The pH of the acid mixture was set to 4.5 with the introduction of pre-standardized 1N NaOH, with subsequent addition of alginic acid in the mixture. The pH of the reaction mixture, under constant stirring, was measured with a pre-calibrated PerpHecT^®^ micro electrode pH meter (ROSS^®^, Thermo Scientific; Waltham, MA, USA) and pH was buffered to 4.5 with the addition of 1N NaOH. The total volume of NaOH added was recorded to adjust the total volume of water used in the formulation development.

### 2.3. Preparation of Acid-Buffered Gel

To develop an acid-buffered gel, the xanthan gum was used as a gelling agent [48,49]. The gel base was prepared by overnight soaking of the pre-weighed amount of xanthan gum in 7 mL MilliQ water. Then, the acid-buffered mixture was transferred to the gel base. Thereafter, the total water content was adjusted in the gel mixture and stirred gently to achieve a uniform gel formulation.

### 2.4. Characterization of Acid-Buffered Gel

#### 2.4.1. Determination of the Acid-Buffering Capacity of the Gel

The acid-buffering capacity of the developed gel was determined against 1N NaOH solution using an approach adapted from a reported procedure [50], slightly modified. Briefly, 1 g of the developed formulation was added in 10 mL 0.9% (*w*/*v*) normal saline, then titrated against 1N NaOH solution added in 10 µL increments to reach pH 7.0. Following each addition, the pH of the dispersed gel mixture was measured and recorded in triplicate. Titration curves were then generated by plotting the average pH against the amount of NaOH added. The data were imported into TableCurve 2D, an automated curve-fitting program, and analyzed to best fit the data using the program’s built-in function, and a sigmoidal shape was generated using the logistic model. The model-generated equation was then used to calculate the amount of NaOH required for changing the pH of the solution to 6 (referred to as the buffering capacity of the gel). The pH of 6 was selected as a measure of determining buffering capacity since a pH above 6 promotes bacterial growth in the wound microenvironment [26].

#### 2.4.2. Dose Determination

**Figure 1:**
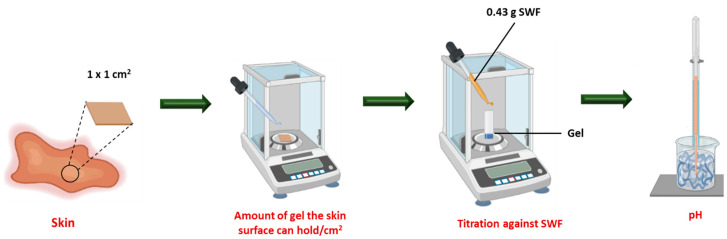
Schematic illustration of the determination of the acid-buffering capacity of gels using SWF (Created in BioRender.com).

The scavenged tissues were collected from humanely killed scavenged mice from the University of South Australia, approved by the Animal Ethics Committee, and preserved at −20 °C. A 1 × 1 cm^2^ tissue section was dissected and used for a dose determination assay. Briefly, 0.50 g of the developed formulation was applied to cover the 1 × 1 cm^2^ tissue section representative of a wound surface as summarized (Figure 1). The weight of the applied gel was recorded and represented as the dose. The determined dose was considered to investigate the acid-buffering capacity of gels against the SWF produced at the rate of 0.43 g/cm^2^/24 h adapted from the published literature [51].

#### 2.4.3. Acid-Buffering Effect Against Simulated Wound Fluid

The acid-buffering capacity was assessed against the SWF at various pH ranges (4, 5.5, 7.7, 8, and 10) corresponding to different physiological conditions of intact skin and infected and non-infected wounds. The SWF was prepared by dissolving MRD (0.95 g) in sterilized MilliQ water (100 mL) and mixing with FBS (100 mL) [29]. The pH of the SWF solution was adjusted with 1N HCl and NaOH, then measured at 12 h and 24 h dosage intervals using a pH meter. The final pH of the gel and its dispersion in SWF were measured and recorded in triplicate.

#### 2.4.4. Viscoelastic Properties

The viscoelastic characteristics of the gels were assessed by means of a Rheosys Merlin VR Rheometer (Scientex Pty Ltd., Melbourne, VIC, Australia) fitted with a parallel plate (15 mm diameter). The experiment was carried out at 25 °C. A sample of both the gel and its dispersion in SWF for each formulation was measured. The oscillatory shear rate sweep and a shear rate of 1–100 s^−1^ were conducted to measure the viscoelastic behavior.

### 2.5. In Vitro Antibacterial Evaluation

#### 2.5.1. Minimum Inhibitory Concentration

The minimum inhibitory concentration (MIC) of the acid-buffered formulations (ABF-1, ABF-2, and ABF-3) was assessed on *S. aureus* (ATCC 19606 (MRSA)), *P. aeruginosa* (PAO1), and *S. epidermidis* (ATCC 35984) using the microdilution method following the Clinical and Laboratory Standards Institute guidelines [52]. The test organisms, retrieved from frozen stock (stored at −80 °C), were grown on TSA plates. The preparation of overnight bacterial cultures was performed by inoculating a single colony into a selective TSB medium and incubating at 37 °C for 24 h. Using the 96-well plates, the individual treatments (ABF-1, ABF-2, ABF-3, and control antibiotic) and TSB were mixed at equal volumes to obtain a 2-fold dilution across the test plates. Afterward, 100 μL of bacterial suspension was dispensed at a final 1 × 10^6^ colony forming units (CFUs/mL) concentration into each well and incubated for 24 h. The initial absorbance at time 0 was recorded using a microplate reader (ELx800 Microplate Reader, BioTek, Winooski, VT, USA) at 620 nm, and then the plates were returned for incubation at 37 °C for 24 h on a shaking incubator. After the 24 h incubation period, the MIC was determined.

#### 2.5.2. In Vitro Wound Biofilm Model

The wound biofilm model was employed to examine the antimicrobial effects of ABF-1, ABF-2, and ABF-3 following a standardized protocol [53,54,55]. In brief, a 25 mm polycarbonate membrane was sterilized with UV light for 15 min and subsequently positioned on BHI agar. The membrane surface was uniformly layered with 30 μL of artificial wound fluid, made from FCS and peptone water (1%), and left to dry. Afterward, 50 μL of 1 × 10^5^ CFU/mL *P. aeruginosa* and *S. aureus* were individually spotted in the centre of each membrane and allowed to incubate for 24 h at 37 °C to enable the development of mature biofilms. After a 24 h period, the membranes were placed on new BHI agar, treated with 100 μL of formulations (ABF-1, ABF-2, and ABF-3), and a control (kanamycin 50 μg/mL), and incubated for another 24 h. The membrane was then transferred to PBS (5 mL) and vortexed for 5 min (twice) followed by twice 15 min sonication to detach the biofilm from the membrane. The suspended bacteria were serially diluted using PBS, plated on TSA, and incubated at 37 °C for 24 h. To quantify the CFUs present in the biofilm, standard colony counts were measured.

### 2.6. In Vitro Cytotoxicity Assay

The cell viability was assessed using an MTT assay [56] to determine the effect of acid-buffered gels on human foreskin fibroblasts (HFFs) and keratinocytes (HaCaT). Both cells were seeded separately in T75 (HFF) and T25 (HaCaT) sterile cell culture flasks using high glucose DMEM containing penicillin and streptomycin (1%), L-glutamine (1%), FBS (15%), HEPES (1.5%), and NEAA (1%). The flasks were incubated under 5% CO_2_ at 37 °C for 24 h. Once the cells reached 90% confluency, the cells were plated on sterile 96-well plates using 100 μL cell media and incubated for another 24 h. After 24 h, the treatments were added (100 μL) and incubated for another 24 h. Afterwards, the cells were gently rinsed twice with PBS and 10 μL of MTT solution was then added. The cells were allowed to incubate for 4 h at 37 °C. Thereafter, the MTT solution was discarded from each well and the MTT formazan crystals were dissolved using DMSO. The dye intensity was measured at 540 nm using the PerkinElmer Wallac microplate plate reader (Waltham, MA, USA).

### 2.7. In Vitro Wound Scratch Assay

To study cell migration, the in vitro wound scratch assay was employed to evaluate the effects of treatments on human skin cell migration [54,57,58]. A 96-well plate was used to seed HFFs and HaCaT at 2 × 10^5^ cells/well and allowed to incubate for 24 h to reach confluency. Using the Incucyte WoundMaker tool (Sartorius, Göttingen, Germany), wounds were created on a single layer of cells, and cells were washed with PBS before adding the treatments to the well. The migration of cells across the wound bed was imaged every 6 h for 36 h with the Olympus Microscope (IX83 Fluorescence Olympus, Tokyo, Japan). The gap between cell fronts was quantified with ImageProPlus 7.0 program (Media Cybernetics Inc., Bethesda, MD, USA), to calculate the percentage of wound closure.

### 2.8. Statistical Analysis

The experiments were performed in triplicate and results were presented as the mean ± standard deviation (SD). Data analysis was performed using either one-way or two-way analysis of variance (ANOVA) to determine significance, with a *p*-value of less than 0.05 indicating significant differences between the control and treatment groups, using GraphPad Prism software (version 10.1.2, San Diego, CA, USA). *, **, ***, and **** indicate *p* < 0.05, *p* < 0.01, *p* < 0.001, and *p* < 0.0001, respectively.

## 3. Results and Discussion

### 3.1. Preparation and Characterization of Acid-Buffered Formulation

#### 3.1.1. The Acid-Buffering Capacity of the Developed Gels

**Figure 2:**
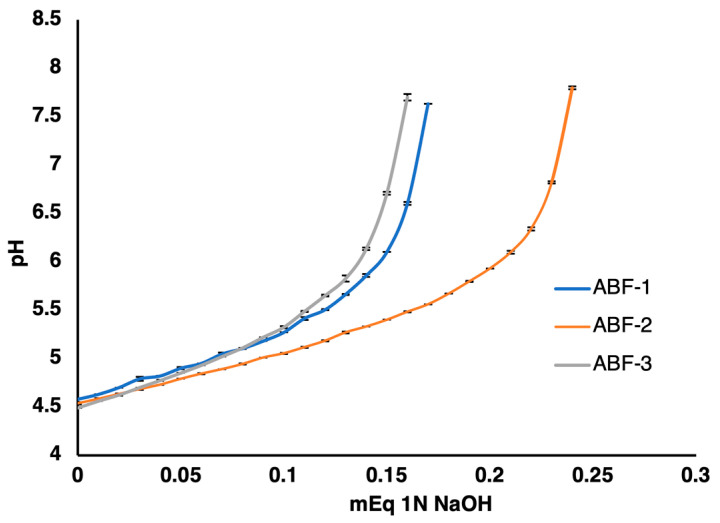
The buffering activity of ABF-1, ABF-2, and ABF-3 against 1N NaOH solution. The results are presented as mean ± standard deviation, *n* = 3.

The acid-buffering capacity of the gels was examined using a 1N NaOH solution. The titration profile of the gels plotted against NaOH is displayed in Figure 2, demonstrating that ABF-2 had the highest buffering capacity compared to formulations ABF-1 and ABF-3. It was observed that formulation ABF-1 required 0.20 mEq of NaOH, whereas formulations ABF-2 and ABF-3 required 0.14 mEq and 0.15 mEq of NaOH, respectively, to change the pH of 1 g of gel from 4.5 to 6.0. These differences in the buffering capacities could be explained by the individual contribution of acids present in each buffered formulation. The three formulations had different acetic acid and citric acid concentrations, while the boric acid concentration was kept constant. Citric acid is a tricarboxylic acid with excellent buffering capacities [59,60], exhibiting three pKa values of 3.13, 4.76, and 6.40, and therefore buffers more effectively over a wider pH range (2.5–7) [61]. In comparison, acetic acid, which is a monoprotic acid with a narrow buffering range, primarily buffers close to its pKa of 4.76 and is therefore relatively effective in the pH range of 3.76 to 5.76 [62]. For this reason, the ABF-2 formulation had the highest buffering capacity since it contained the highest amount of citric acid, while formulation ABF-1 had a low citric acid amount and its acetic acid concentration was slightly higher, which yielded an overall low buffering capacity compared to the ABF-2 formulation. The ABF-3 formulation was composed of the least amount of both acids resulting in the lowest buffering capacity.

**Figure 3:**
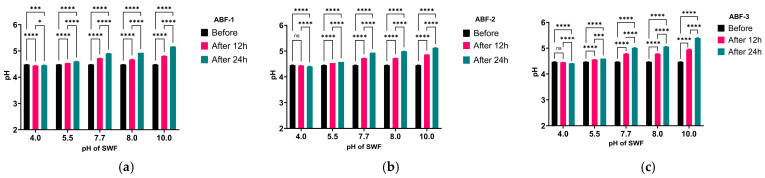
The buffering activity of (**a**) ABF-1, (**b**) ABF-2, and (**c**) ABF-3 against SWF at different pH ranges where ‘before’ indicates the pH of the formulation before the start of titration. The results are presented as mean ± standard deviation, *n* = 3. * shows a significant difference compared to control (*p* < 0.05) using two-way ANOVA, where ns = non-significant, *** = *p* < 0.001 and **** = *p* < 0.0001.

Additionally, to study the clinically relevant buffering capacity of the gels, the SWF wound was used and adjusted at different pH ranges representing the pH of healthy intact skin, and the acute and chronic wound pHs of both infected and non-infected wounds. The study was carried out to determine any disruptions in the formulations’ buffering capacities when titrated against SWF [29]. The initial pH of the SWF was recorded to determine pH changes after 12 h and 24 h dosage intervals when titrated with the gels (Figure 3). The results showed that all formulations exhibit excellent buffering capacities at all pH ranges, with the maximum change in pH found after titration with SWF of pH 10, with the initial pH of ABF-1—SWF dispersion rising from 4.47 ± 0.01 to 4.81 ± 0.01 at a 12 h dosage interval and 5.16 ± 0.01 after a 24 h dosage interval (Figure 3a), while ABF-2 had a change from an initial pH of 4.47 ± 0.01 to a pH of 4.87 ± 0.01 after a 12 h dosage interval and 5.12 ± 0.01 after a 24 h dosage interval (Figure 3b) (Table 2). Additionally, ABF-3 displayed a change in pH from 4.48 ± 0.01 to 4.95 ± 0.02 and 5.39 ± 0.02 after 12 h and 24 h dosage intervals, respectively, when titrated against SWF at pH 10 (Figure 3c) (Table 2). These maximum final pH values of gel–SWF dispersions indicate that the formulations have the strong capacity to maintain an acidic pH that is favorable for wound healing and inhibiting microbial growth [33]. Moreover, the resulting pH indicates the ability of the gels to be developed as effective topical formulations that require a pH ranging between 4.2 and 5.6 [63,64].

#### 3.1.2. Assessing Rheological Properties

**Figure 4:**
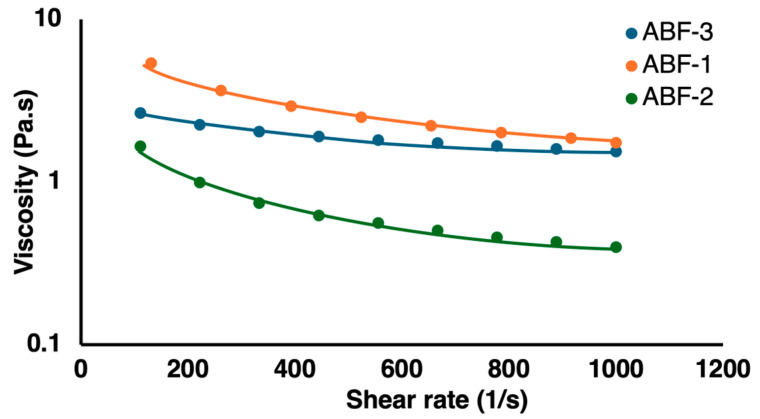
Rheological properties of acid-buffered gels measured by shear rate after the equilibrium state at 25 °C.

The viscoelastic behavior of the acid-buffered gels was studied to assess the rheological properties. As shown in Figure 4, the gels exhibited shear thinning behavior, which was explained by their reduced viscosity as the shear rate increased. The observed starting viscosity in all three formulations was different because of the composition, but the shear thinning behavior was almost similar. This behavior is attributed to the formation of intermolecular interactions and crosslinking between the hydroxyl and carboxyl groups of alginic acid and xanthan gum, creating strong hydrogen bonding, thereby resulting in the increased viscosity and improved physical strength of the gels [48]. This characteristic is important to manage the flow of wound exudates and allow the filling of irregularly shaped wounds on topical application [10,33].

### 3.2. Evaluation of In Vitro Antibacterial Activity

#### 3.2.1. In Vitro Inhibition of Bacterial Growth Using Acid-Buffered Gels

The effect of organic acids including citric acid [29,36,37,39,43,65,66,67,68], boric acid [69,70], and acetic acid [40,42,44,45,71] in inhibiting bacterial growth has been widely investigated previously against Gram-positive and Gram-negative bacterial species in chronic wounds, showing promising antibacterial effects. In this study, the in vitro antibacterial effect of acid-buffered gels composed of a combination of organic acids, namely citric acid, boric acid, and acetic acid, along with alginic acid, was tested against *S. aureus*, *S epidermidis*, and *P. aeruginosa*, and compared against an antibiotic (kanamycin) using the micro-broth dilution method [53]. Our results revealed that all three formulations possessed excellent antimicrobial activities, with ABF-1 and ABF-2 exhibiting a slightly high MIC at one-eighth dilution compared to ABF-3 which exhibited an inhibitory effect on bacterial growth at one-quarter dilution against Gram-positive strains (Table 3). The MIC against Gram-negative bacteria was, however, comparable and equal for all three formulations. This slight difference observed in the MICs of the three acid-buffered formulations is explained by the difference in the total ionic strengths of the organic acids in the formulations as described previously in Section 3.1.1.

The inhibitory effect on bacterial growth could be explained by the differing mechanisms contributed by individual acids and the overall low pH of the buffered formulations. Firstly, the acids create a low-pH microenvironment around the wound bed that regulates numerous cellular processes, such as the migration of macrophages and enzymatic activities, and makes unfavorable growth conditions for bacteria [29,33,72,73]. Secondly, the antibacterial action of organic acids involves acidification of bacterial cytoplasm through their accumulation at toxic concentrations as dissociated anions, which further disrupts bacterial metabolic pathways. The strength of antibacterial activity depends on the concentration of acids and the degree of dissociation of individual acids. At a low pH, the dissociation of acids is enhanced, further promoting their diffusion across microbial cellular membranes and thereby leading to antibacterial effects [65,74,75]. Thirdly, alginic acid contributes to the antibacterial effect through multiple mechanisms. With its fluid-absorbing abilities, alginic acid absorbs wound exudates, which creates an environment unfavorable for bacterial proliferation [76]. In addition to this, the negative charge found on alginates allows alginic acid to attach to the outer cellular surfaces of bacteria, thereby disrupting the membrane that leads to the seepage of intracellular components [77,78,79,80]. Additionally, the attachment of alginic acid to bacterial membranes creates a viscous layer around bacteria that prevents the exchange of nutrients across the membrane [78]. Moreover, another mechanism by which alginic acid could produce an antibacterial effect is through its chelating properties that modulate toxin production which disrupts bacterial mechanisms [79]. This effect is related to its M-block content, which yields immunostimulant properties, causing the activation of macrophages and eventual cytotoxin productions that damages microbes [78,81,82].

These in vitro results indicate that our acid-buffered formulations exhibit high antimicrobial efficacy against both Gram-positive and Gram-negative species, with the incorporation of citric acid, and acetic acids in high concentration, aiding in better antimicrobial properties.

#### 3.2.2. In Vitro Bacterial Wound Biofilm Activity of Acid-Buffered Gels

**Figure 5:**
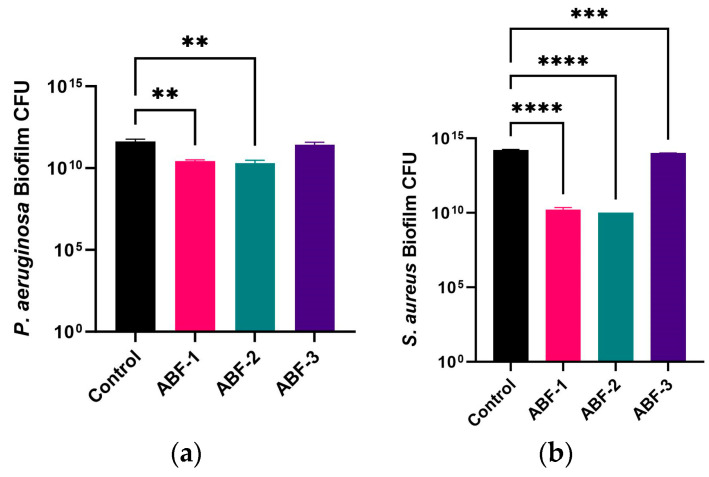
Wound biofilm assay (**a**) *P. aeruginosa* and (**b**) *S. aureus*. The results are displayed as mean ± standard deviation, *n* = 3. ** = *p* < 0.01, *** = *p* < 0.001, and **** = *p* < 0.0001.

The in vitro anti-biofilm effect of the acid-buffered formulations was investigated using bacterial attachment assays where the CFU method was used to quantify biofilms. The results revealed that all formulations significantly reduced the bacterial attachment when tested against *P. aeruginosa*, with ABF-1 and ABF-2 exhibiting the most pronounced effect as compared to ABF-3, which showed comparatively less reduction in the bacterial CFUs (Figure 5a). In contrast to this, a significant reduction in bacterial attachment was also observed against *S. aureus* biofilms; however, this effect was less pronounced than *P. aeruginosa* biofilms’ eradication (Figure 5b). This effect was consistent with the previous observations which showed that complete eradication of the *P. aeruginosa* biofilms was achieved when tested using acetic acid compared to *S. aureus* biofilms, which required higher concentrations of the acid to achieve similar effects [35]. Additionally, the differences in the effect of ABF-3 compared to ABF-1 and ABF-2 are further explained by the total ionic strengths of acids in the formulations, with ABF-1 and ABF-2 exhibiting a pronounced effect on reducing bacterial load.

Previous studies examining the mechanism of biofilm eradication using acids have proposed several mechanisms that could contribute to the reduction in bacterial biofilms. Firstly, the effect is not caused by the pH of the acids alone, previously confirmed by testing solutions of matching pH using both organic and inorganic acids, indicating that the same pH of different acids produced different results [34,35]. Rather, the effect is a consequence of organic acids’ ability to cross the cell membranes of bacteria and readily dissociate into ionized and non-ionized states, which disturbs the ionic gradient and the intracellular pH, disrupting cellular processes and eventual cell death [34,35,83,84,85,86,87,88].

### 3.3. In Vitro Assessment of Cytotoxicity Study and Wound Healing Properties

**Figure 6:**
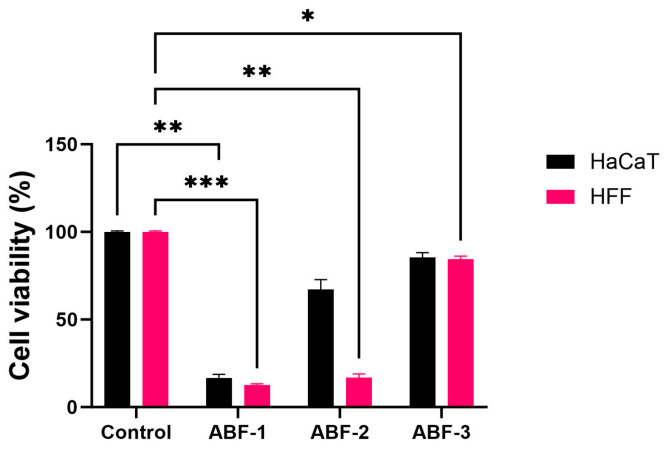
In vitro cell viability analysis in response to treatment with acid-buffered gels in healthy fibroblast and keratinocyte cells. The results are expressed as mean ± standard deviation (*n* = 3). * Shows a significant difference compared to control (*p* < 0.05) using two-way ANOVA. ** *p* < 0.01, *** *p* < 0.001.

**Figure 7:**
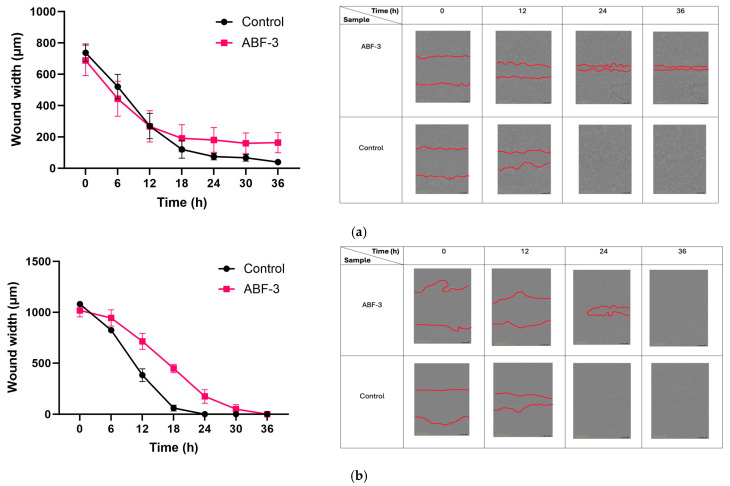
Measurement of wound closure (measured as wound width) and representative images demonstrating the migration of (**a**) HaCaT and (**b**) HFF cell lines after the treatment with ABF-3. The error bar represents the mean ± standard deviation (*n* = 3).

The healthy skin cells (HFF and HaCaT) were used to determine the in vitro biocompatibility of acid-buffered gels by studying the effects on healthy cell function and wound healing response. The in vitro cytotoxicity test was conducted to measure the metabolic activity of the cell lines in response to the formulation treatment after 24 h. The results showed that ABF-1 and ABF-2 significantly reduced the viability of the fibroblasts and keratinocytes (Figure 6). This is a result of high acid strengths in ABF-1 and ABF-2, significantly impacting the viability of healthy cells, confirmed through previous studies where high ionic strength acid buffers have induced apoptosis of keratinocyte and fibroblast cells [27,89,90,91,92]. This effect is caused by the formation of crystals in the cellular media as a result of ionic disruptions contributed by high concentrations of acids [90,92]. Interestingly, ABF-3 had a minor effect on the viability of the cells, with more than 80% viability of both cell lines (Figure 6). The increasing viability is due to the low ionic concentration of the treatment compared to ABF-1 and ABF-2. Additionally, a similar effect has been reported by Sim et al. (2022) where low ionic strength acidic buffers showed positive cell viability and also demonstrated enhanced cell growth [92]. Based on this study, ABF-3 was considered for further biocompatibility studies using a wound scratch assay. The results revealed that ABF-3 demonstrated no negative impact on fibroblast and keratinocyte cell migration (Figure 7a). The wound closure measurement was comparable to the control when measured at various time points of treatment for 36 h (Figure 7b). The results of this study indicate that the acid-buffered treatments of relatively low ionic strength are safe to apply to treat bacterial infections without significantly hindering the cellular responses that are essential for tissue regeneration.

## 4. Conclusions

In this study, a novel acid-buffered gel was developed and characterized using a combination of organic acids with alginic acid and xanthan gum to treat wound infections. By optimizing the acidic pH buffered at 4.5 within the wound environment, this formulation aims to offer an effective alternative to traditional antibiotics, providing a crucial tool in the fight against wound infections in the era of escalating antimicrobial resistance. The acid-buffered formulation demonstrated promising in vitro antibacterial activity when tested against *S. aureus*, *S. epidermidis*, and *P. aeruginosa*, showing inhibition of bacterial growth. Additionally, the gels composed of high acid concentrations demonstrated promising effects in eliminating the bacterial load of *S. aureus* and *P. aeruginosa* chronic wound biofilms and possessed potential acid-buffering capacities when titrated against simulated wound fluid (SWF) at different pH values representing the wound microenvironments of infected and non-infected wounds. Furthermore, the gel with low acid strength positively affected the viability of keratinocytes and fibroblasts and improved wound closure, signifying its potential for promoting wound healing. The presented study demonstrates that the acid-buffered gel with optimized acid strengths offers a novel topical treatment for chronically infected wounds. The developed formulation warrants further in vivo mouse wound infection studies to determine its efficacy and safety as a potential antibacterial formulation to combat wound infections.

## Figures and Tables

**Table 1:** Composition of acid-buffered gels.

Formulation	Citric Acid	Acetic Acid	Boric Acid	Alginic Acid
ABF-1	1%	1.5%	1%	4%
ABF-2	1.5%	1%	1%	4%
ABF-3	1%	1%	1%	4%

**Table 2:** The buffering capacity of acid-buffered formulations when titrated against SWF of different pHs.

SWF pH	pH Before Titration			pH After Titration (12 h)			pH After Titration (24 h)		
	ABF-1	ABF-2	ABF-3	ABF-1	ABF-2	ABF-3	ABF-1	ABF-2	ABF-3
4	4.47 ± 0.02	4.47 ± 0.01	4.48 ± 0.01	4.44 ± 0.01	4.45 ± 0.01	4.46 ± 0.01	4.46 ± 0.01	4.41 ± 0.02	4.41 ± 0.01
5.5	4.47 ± 0.01	4.47 ± 0.01	4.48 ± 0.01	4.53 ± 0.01	4.54 ± 0.00	4.56 ± 0.01	4.6 ± 0.01	4.58 ± 0.00	4.60 ± 0.00
7.5	4.47 ± 0.01	4.47 ± 0.01	4.48 ± 0.01	4.72 ± 0.01	4.73 ± 0.01	4.79 ± 0.01	4.91 ± 0.01	4.94 ± 0.00	5.01 ± 0.01
8	4.47 ± 0.01	4.47 ± 0.01	4.48 ± 0.01	4.67 ± 0.02	4.73 ± 0.01	4.79 ± 0.01	4.93 ± 0.00	4.99 ± 0.01	5.05 ± 0.02
10	4.47 ± 0.01	4.47 ± 0.01	4.48 ± 0.01	4.81 ± 0.01	4.87 ± 0.01	4.95 ± 0.02	5.16 ± 0.01	5.12 ± 0.01	5.39 ± 0.02

**Table 3:** MIC of acid-buffered formulations against *S. aureus*, *S. epidermidis*, and *P. aeruginosa*.

Treatments	*S. aureus* (Dilution)	*S. epidermidis* (Dilution)	*P. aeruginosa* (Dilution)
ABF-1	1/8	1/8	1/8
ABF-2	1/8	1/8	1/8
ABF-3	1/4	1/4	1/8
Control (Kanamycin)	6400 μg/mL		

## Data Availability

Data available on request from co-authors.
